# Food-specific IgG antibodies and body mass index: multivariate analysis of clinical correlations in underweight populations

**DOI:** 10.3389/fimmu.2025.1650705

**Published:** 2025-10-21

**Authors:** Yao-Chi Zeng, Cui-Yu Li, Xiao-Li Song, Shu-Fen Huang, Yi Xie, Juan Zeng, Rui Yuan

**Affiliations:** ^1^ Department of Clinical Nutrition, Shenzhen Traditional Chinese Medicine Hospital, Shenzhen, China; ^2^ Department of Health Education, Shenzhen Traditional Chinese Medicine Hospital, Shenzhen, China; ^3^ Department of Infection Control, Shenzhen Traditional Chinese Medicine Hospital, Shenzhen, China; ^4^ Department of Central Sterile Supply, Shenzhen Traditional Chinese Medicine Hospital, Shenzhen, China; ^5^ Department of Clinical Laboratory, Shenzhen Traditional Chinese Medicine Hospital, Shenzhen, China

**Keywords:** food intolerance, IgG antibodies, body mass index, underweight, chronic low-grade inflammation, nutritional biomarkers, cluster analysis, personalized dietary interventions

## Abstract

**Background:**

This study investigates the relationship between food-specific IgG antibodies and nutritional status in underweight populations, addressing a critical gap in existing research focused predominantly on obesity. It aims to elucidate immune-mediated mechanisms linking food intolerance to abnormal body composition through multidimensional statistical modeling.

**Methods:**

A retrospective analysis of 1,237 underweight patients (BMI <18.5 kg/m²) included IgG antibody profiling for 14 food antigens (ELISA) and clinical/demographic data. Statistical methods encompassed Spearman correlations, linear regression, factor analysis, and generalized linear models (adjusted for age, gender, comorbidities). Child (n=421) and adult (n=816) cohorts were analyzed separately using R 4.3.0 and GraphPad Prism 9.0.

**Results:**

In children, wheat-specific IgG levels showed a robust inverse correlation with BMI-for-age Z-scores (BAZ) (β = -0.319 to -0.357, p ≤ 0.010), explaining 2.18% of BAZ variance. Factor analysis identified a food sensitivity component (wheat/soy IgG loadings: 0.643–0.654) correlating with BAZ (r = 0.349). Adults exhibited significant inverse associations between soybean IgG and BMI (β = -1.1085, p = 0.0003), explaining 1.67% of variance. Bilirubin metabolism (factor loadings: 0.899–0.991) and hepatic function markers (ALT/GGT: r = 0.372–0.425) showed strong BMI correlations. Cluster analysis revealed distinct IgG profiles, with underweight subgroups demonstrating elevated wheat (p = 0.001) and soybean (OR = 2.4, p < 0.001) sensitization.

**Conclusion:**

Food-specific IgG profiles, particularly wheat and soybean antibodies, are independently associated with nutritional status in underweight populations. These findings suggest immune-mediated pathways may contribute to malabsorption and metabolic dysregulation, supporting IgG testing for personalized dietary interventions. Study limitations include small subgroup sizes, underscoring the need for mechanistic research integrating gut microbiota analysis.

## 1 Introduction

The role of immunoglobulin G (IgG) antibodies in mediating food-related immune responses has garnered increasing attention in nutritional science, particularly concerning metabolic dysregulation (1, 2). Food-specific IgG antibodies, distinct from IgE-mediated allergic reactions, are implicated in delayed immune responses to dietary antigens, potentially contributing to chronic inflammation and impaired nutrient absorption (3). While substantial evidence links elevated IgG levels to obesity-related metabolic disturbances—including insulin resistance and dyslipidemia (4)—research remains disproportionately focused on overweight populations, with clinical protocols often prioritizing IgG testing for weight management interventions (5). Recent investigations have revealed that IgG specifically accumulates in adipose tissue of obese individuals, not only inducing local inflammation but also directly interfering with insulin receptor binding via its Fc-CH3 domain, thereby impairing adipogenesis and metabolic function (6).

This obesity-centric paradigm overlooks the potential bidirectional relationships between IgG profiles and nutritional status across the body mass index (BMI) spectrum, particularly in underweight individuals where immune-mediated malabsorption may exacerbate energy deficits.

Underweight status (BMI <18.5 kg/m²) affects over 462 million adults globally, with disproportionate prevalence in low-income regions and populations with chronic comorbidities (7). It is important to note that the role of food-specific IgG, particularly the IgG4 subclass, remains controversial: it may represent a normal adaptive immune response to persistent dietary antigen exposure, promoting regulatory T-cell-mediated tolerance; conversely, elevated food-specific IgG levels have also been associated with increased intestinal permeability and inflammatory conditions, as observed in eosinophilic esophagitis, irritable bowel syndrome, and related disorders (8).

Despite established associations between IgG-mediated food intolerance and gastrointestinal dysfunction, mechanistic studies examining immune-nutrition interactions in undernutrition remain scarce (9). Emerging evidence suggests that persistent IgG reactivity to common dietary antigens, such as wheat and soy, may impair intestinal barrier integrity, potentially disrupting micronutrient absorption and hepatic metabolism (10). Studies have identified that humans naturally harbor circulating IgG antibodies reactive to peptide hormones (such as ghrelin), which may function as carrier molecules that protect hormones from degradation and modulate their receptor binding, suggesting complex regulatory roles for IgG across different nutritional states (11). However, critical gaps persist in understanding whether elevated IgG titers in underweight individuals represent adaptive responses to nutrient deprivation or pathogenic drivers of metabolic dysregulation, limiting the development of targeted nutritional therapies.

This study addresses the obesity-centric bias in IgG research by investigating immune-mediated pathways linking food-specific IgG profiles to abnormal body composition in underweight populations. Building on prior work demonstrating IgG-guided dietary efficacy in irritable bowel syndrome (12), we hypothesize that specific IgG patterns may serve as biomarkers for immune-mediated malabsorption. ​It is worth emphasizing that current diagnostic guidelines from major allergy and immunology societies do not recommend routine use of IgG testing for diagnosing food allergies and intolerances, as these antibodies may indicate exposure or underlying inflammation rather than causative mechanisms; however, this study aims to further clarify their clinical relevance through more refined design (8).​Our analysis extends beyond univariate associations to model multifactorial interactions between IgG reactivity, hepatic function, and nutritional biomarkers—a novel approach bridging immunology and clinical nutrition.

Our investigation focuses on a cohort from Shenzhen, China (2022–2023), a characteristic immigrant city with a culturally diverse population and consequently highly integrated dietary habits, incorporating Cantonese, Hunanese, Sichuanese, and other regional culinary traditions (13).

Methodologically, we employ retrospective cohort analysis with pediatric-adult stratification to account for developmental variations in immune responses and nutritional requirements. Advanced statistical modeling integrates IgG profiling against 14 food antigens with clinical covariates, enabling detection of subtle immune-metabolic interactions often obscured in smaller studies. This approach builds upon methodologies validated in obesity research (14) while introducing age-specific adjustments for growth parameters and comorbidity profiles.

The identification of wheat and soybean IgG as independent predictors of nutritional status holds significant clinical implications. These findings align with proteomic studies identifying wheat-derived peptides as triggers of intestinal immune activation (4), suggesting IgG testing could personalize dietary interventions for underweight patients. Furthermore, observed correlations between IgG profiles and hepatic markers extend current models of the gut-liver axis, complementing emerging research on microbiota-immune interactions in malnutrition (15). By establishing IgG as a modifiable factor in undernutrition, this work advances precision nutrition strategies while highlighting the need for longitudinal studies integrating microbiome analysis and metabolomic profiling (16, 17).

## 2 Methods

### 2.1 Study population and data collection

This retrospective study analyzed data from 1,280 patients with recorded BMI measurements who received treatment between January 2022 and December 2023. Data collected from electronic medical records included patient demographics (age, sex, geographic region) and clinical history (documented comorbidities and duration of underweight status). The analysis included a wide range of clinical parameters: metabolic assessments of liver and renal function, blood biochemistry profiles, lipid and glucose metabolism indicators, complete blood count, immune system markers, vitamin levels, thyroid function tests, and muscle enzyme measurements. Food-specific IgG antibodies were tested for common dietary components including milk, eggs, wheat, rice, corn, soy, seafood (cod, crab, shrimp), and meats (beef, pork, chicken). All biochemical measurements were evaluated against established reference ranges and classified as low, normal, or high based on standardized thresholds.

### 2.2 Laboratory analyses

Food-specific IgG antibodies for milk, egg, wheat, rice, corn, soybean, tomato, cod, crab, mushroom, shrimp, beef, pork, and chicken were all determined using enzyme-linked immunosorbent assay (ELISA) kits (manufacturer details withheld for anonymization) following the standardized protocol described by Patil et al. (18). Serum samples underwent a four-step process: 1) antigen-coated plate incubation (2 hours, 37°C), 2) blocking with 5% bovine serum albumin (30 minutes, room temperature), 3) serum sample incubation (1:100 dilution, 1 hour, 37°C), and 4) horseradish peroxidase-conjugated secondary antibody detection (45 minutes, 37°C). Optical density values were measured at 450 nm using a microplate reader, with results expressed as kilounits per liter (kU/L). Positive thresholds followed manufacturer-defined cutoffs (>12 kU/L for all antigens). Except, the following indicators were systematically assessed in this study. Basic demographic information, including age group and gender, was collected through standardized questionnaires. BMI was calculated from objectively measured height and weight. Liver function parameters, including alanine aminotransferase (ALT), aspartate aminotransferase (AST), gamma-glutamyl transferase (GGT), total bilirubin (TB), direct bilirubin (DB), and indirect bilirubin (IB), were analyzed using an automated biochemical analyzer. Renal function was assessed by measuring urea, creatinine (CREA), and uric acid (UA) with the same automated biochemical analyzer, while cystatin C (CysC) was determined by immunoturbidimetric assay. The glomerular filtration rate (GFR) was estimated based on CREA levels. Protein nutritional status indicators, including total protein (TP), albumin (ALB), globulin (GLB), and prealbumin (PA), were measured using an automated biochemical analyzer, with PA quantified via immunoturbidimetry. Vitamin status was evaluated by measuring 25-hydroxyvitamin D using a chemiluminescence immunoassay, and vitamins K1 and K2 were assessed using enzyme-linked immunosorbent assay (ELISA).

### 2.3 Statistical analysis

#### 2.3.1 Descriptive statistics

The IgG-positive rate was calculated as the percentage of patients exceeding the diagnostic threshold for each antigen. BMI distribution was characterized using median, interquartile range (IQR), and 95% confidence intervals (CIs). Continuous variables were tested for normality via Shapiro-Wilk tests (α = 0.05).

#### 2.3.2 Correlation analysis

Spearman’s rank-order correlation coefficient (ρ) quantified associations between IgG antibody titers (log10-transformed to normalize skewness) and BMI values. Nonparametric methods were selected due to non-normal distributions of IgG titers (p < 0.001 for Kolmogorov-Smirnov tests). Significance thresholds were adjusted using Benjamini-Hochberg correction for multiple comparisons across 14 antigens.

#### 2.3.3 Factor analysis

Principal component analysis (PCA) reduced dimensionality in the IgG antibody dataset (14 variables). Components with eigenvalues >1.0 were retained based on Kaiser’s criterion. Varimax rotation optimized interpretability of factor loadings. IgG titers were z-score standardized prior to PCA to eliminate scale-dependent biases.

### 2.4 Regression modeling

The analysis employed two multivariate models. A linear regression model used BMI as the dependent variable with log10-transformed IgG titers as primary predictors. Additionally, a generalized linear model was developed with adjustments for age (continuous), sex (categorical: male/female/other), and comorbidity burden (ordinal: 0, 1–2, ≥3 conditions). Variance inflation factors (VIFs) were used to assess multicollinearity with a threshold of VIF <5).

### 2.5 Computational tools and software

Analyses were conducted in R 4.3.0 (R Foundation for Statistical Computing) using the *stats* package for regression and PCA, and *factoextra* for cluster visualization.

## 3 Results

### 3.1 Cohort establishment and data quality control

Systematic data cleaning of 1,280 initial records yielded 1,237 analyzable cases after excluding 43 participants below age 5. The final cohort comprised 421 children (5–18 years) and 816 adults (>18 years), with complete age and BMI data retained per study protocol. Pediatric participants included 144 underweight cases (BAZ < −1 + clinical diagnosis) versus 277 non-underweight controls (**Supplementary Table 1**), while adults contained 218 underweight cases (BMI < 18.5 + diagnosis) versus 598 non-underweight controls (**Supplementary Table 2**). Gender distribution revealed a predominantly male pediatric cohort (54%) contrasting with a female-dominated adult population (74.6%) (**Supplementary Table 1**, **2**).we next investigated whether nutritional biomarkers could better differentiate clinical subgroups.

### 3.2 Biomarker profiles and food sensitivity patterns in children

Vitamin K2 sufficiency differed significantly between groups (94.6% nutrition problem vs. 89.5% controls, *p* = 0.013), while vitamin D deficiency rates inversely varied (10.8% vs. 23.6%, *p* = 0.017, **Table 1**). Food-specific IgG analysis revealed marked wheat sensitization differences (*p* = 0.001), with the nutrition problem group showing higher grade 2 (22.9% vs. 15.3%) and grade 3 (10.4% vs. 2.9%) responses (**Table 1**). Mushroom IgG responses showed borderline significance (*p* = 0.049), while other antigens demonstrated no significant between-group variation (**Table 1**). To elucidate the interplay between these biomarkers and anthropometric measures, we conducted multivariate factor analysis. To establish baseline characteristics for subsequent analyses, we next analyzed population demographics and symptom profiles across age groups.

**Table 1:** Comparison of significant baseline characteristics between children with and without underweight status.

indicator	Non-wasting (n=277)	Wasting (n=144)	p-value
Sex, Female (n, %)	118 (42.6%)	71 (49.3%)	0.227
Age Group (n, %)			0.081
5–10 years	88 (31.8%)	60 (41.7%)	
10–15 years	155 (56.0%)	73 (50.7%)	
15–18 years	34 (12.3%)	11 (7.6%)	
BAZ (mean, SD)	0.92 (1.99)	-1.57 (0.39)	**<0.001**
GGT Status (n, %)			**0.018**
Low	5 (10.4%)	4 (50.0%)	
Normal	42 (87.5%)	4 (50.0%)	
Wheat IgG (n, %)			**0.001**
Level 0 (Negative)	149 (54.2%)	60 (41.7%)	
Level 1 (Weak)	76 (27.6%)	36 (25.0%)	
Level 2 (Moderate)	42 (15.3%)	33 (22.9%)	
Level 3 (Strong)	8 (2.9%)	15 (10.4%)	
Mushroom IgG (n, %)			**0.049**
Level 0 (Negative)	234 (85.1%)	128 (88.9%)	
Level 1 (Weak)	30 (10.9%)	15 (10.4%)	
Level 2 (Moderate)	11 (4.0%)	0 (0.0%)	
Level 3 (Strong)	0 (0.0%)	1 (0.7%)	

Bold values indicate significant p-values (p < 0.05).

### 3.3 Pediatric population characteristics and symptom correlations

The pediatric cohort (median age 8.2 years, IQR 5.4–11.3) exhibited high malnutrition prevalence (49.0%, 201/410) alongside substantial obesity rates (22.7%, 93 patients). Malnutrition (ρ = −0.570, *p* < 0.001) and wasting (ρ = −0.562, *p* < 0.001) demonstrated strong inverse correlations with BAZ, while obesity showed the strongest positive association (ρ = 0.854, *p* < 0.001). Fatty liver disease displayed moderate positive correlation (ρ = 0.408, *p* < 0.001), whereas growth retardation showed weaker negative association (ρ = −0.180, *p* < 0.001). Common symptoms, including allergic rhinitis, constipation, and sleep disorders, showed no significant BAZ relationships after multiple testing correction (**Table 2**).

**Table 2:** Correlation analysis between symptoms and BAZ scores in pediatric population (significant results only).

Symptom	Frequency (n)	Frequency (%)	ρ	p-value
Malnutrition	201	49.00%	-0.57	< 0.001
Wasting	174	42.40%	-0.562	< 0.001
Obesity	93	22.70%	0.854	< 0.001
Growth retardation	51	12.40%	-0.18	< 0.001
Eczema	19	4.60%	0.13	0.009
Fatty liver	18	4.40%	0.408	< 0.001
Hyperuricemia	17	4.10%	0.268	< 0.001
*Allergic rhinitis*	*64*	*15.60%*	*-0.06*	*0.224 (NS)*

### 3.4 Multivariate factor analysis of pediatric metabolic profiles

Pearson correlation analysis revealed significant associations (p < 0.05) between the core indicator (BAZ) and several indicators. A total of 10 indicators showed significant correlation. Detailed correlation coefficients (r) and p-values are presented in **Table 3**.

**Table 3:** Correlation analysis between BAZ and key indicators.

Indicator	Correlation Coefficient	P-Value
Alanine aminotransferase (ALT)	0.425	p<0.001
γ-Glutamyl transferase (GGT)	0.372	p<0.001
Uric acid (UA) (90–420 umol/L)	0.295	p<0.001
Total Vitamin D (VitD-T)	-0.171	p<0.001
Aspartate aminotransferase (AST)	0.164	p<0.001
Prealbumin (PA)	0.149	p=0.002
Wheat-specific IgG antibody	-0.148	p=0.003
25-hydroxyvitamin D	-0.124	p=0.012
Globulin (GLB)	0.114	p=0.020
Total protein (TP)	-0.107	p=0.030

**Figure 1:**
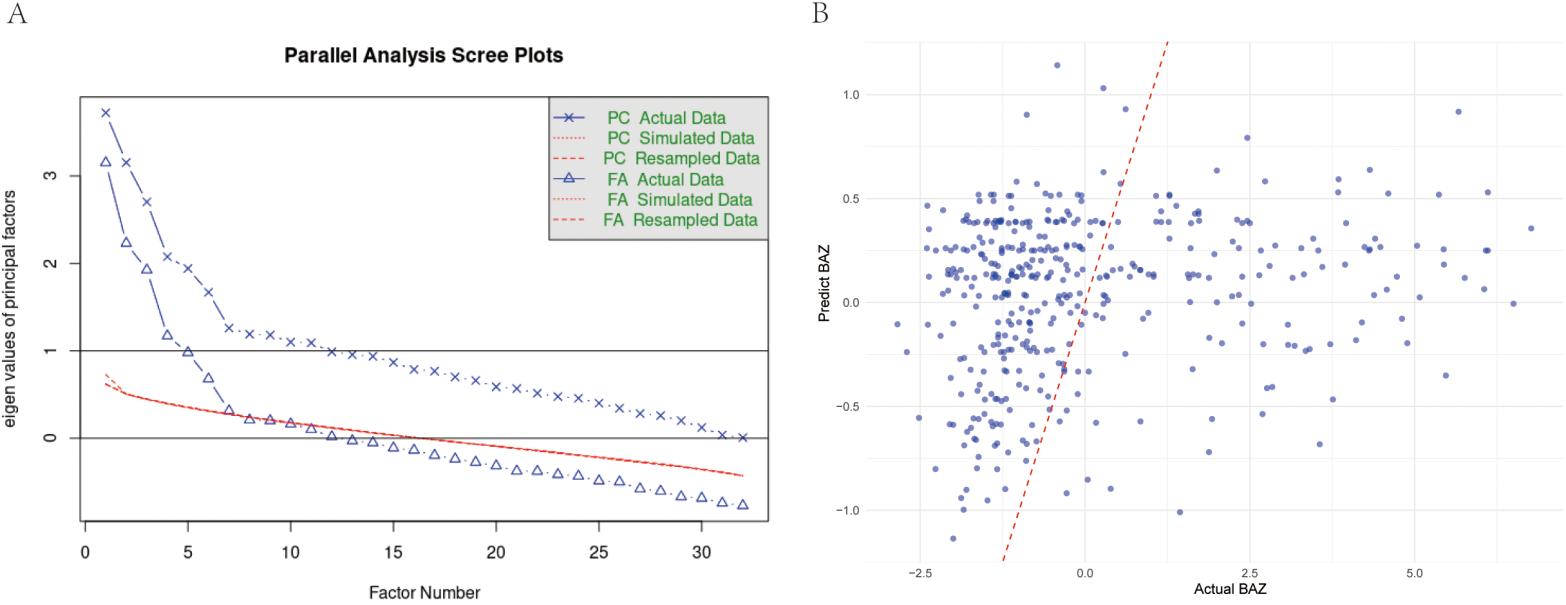
**(A)** Parallel analysis scree plot for all valid indicators. This plot compares eigenvalues from actual data (solid line, dots) with mean eigenvalues generated from random data (dashed line, crosses) to determine the number of factors. The red line represents the eigenvalue threshold of 1 (Kaiser criterion). **(B)** Comparison of predicted vs. actual BAZ values using multivariate GLM model in children group. Scatter plot showing the relationship between model-predicted BAZ values and actual BAZ values, with a red dashed line indicating perfect prediction.

Exploratory Factor Analysis was performed to explore the underlying structure among the indicators significantly correlated with the core indicator (BAZ). Additionally, we conducted factor analysis on all valid indicators to explore broader underlying structures. Seven factors explained 42.4% of total variance (**Figure 1A**). Factor 3 (food sensitivity: wheat IgG loading = 0.643, soy = 0.654) showed the strongest BAZ correlation (*r* = 0.349). The hepatic enzyme factor (ALT = 0.900, AST = 0.827) correlated moderately with BAZ (*r* = 0.372–0.425), while the bilirubin metabolism factor (total bilirubin = 0.991, direct = 0.899) showed no significant association (**Table 4**). Noting the prominent role of food sensitivity in BAZ variation, we performed targeted analysis of antigen-specific IgG associations.

**Table 4:** Factor analysis results and their correlation with BAZ.

Factor	Name & interpretation	Major loading indicators (Loading ≥ |0.4|)	Correlation with BAZ
Factor1(PA1)	Bilirubin Metabolism	Total Bilirubin (0.991)	-0.055
		Direct Bilirubin (0.899)	
		Indirect Bilirubin (0.938)	
Factor2(PA2)	Liver Enzyme Activity	ALT (0.900)	-0.085
		AST (0.827)	
		γ-GGT (0.795)	
Factor3(PA3)	Grain Food Sensitivities	Soy IgG (0.654)	0.349
		Wheat IgG (0.643)	
		Rice IgG (0.597)	
		Corn IgG (0.562)	
Factor4(PA4)	Meat Sensitivities	Beef IgG (0.936)	0.226
		Chicken IgG (0.634)	
		Pork IgG (0.581)	
Factor5(PA5)	Nutrition & Renal Function	Prealbumin (0.713)	-0.038
		Albumin (0.602)	
		Cystatin C (-0.424)	
Factor6(PA6)	Seafood Sensitivities	Crab IgG (0.798)	0.05
		Shrimp IgG (0.723)	
Factor7(PA7)	Serum Protein Level	Globulin (0.645)	0.076
		Total Protein (0.623)	

### 3.5 Dose-dependent association between wheat IgG and pediatric BAZ

Wheat-specific IgG levels demonstrated robust inverse BAZ relationships in univariate (β = −0.319, *p* = 0.003) and multivariate models (β = −0.357, *p* = 0.010), explaining 2.18% of BAZ variance (**Figure 1B**). Each unit increase predicted a 0.32–0.36 SD BAZ decrease. No other food antigens showed significant associations (13 antigens *p* > 0.08) (**Table 5**). Generalized linear model diagnostics confirmed linear fit without plateau effects. To assess whether similar mechanisms operate in adults, we extended our analysis to the older cohort.

**Table 5:** Linear regression analysis of food-specific IgG antibodies and their association with BAZ in pediatric population.

Food-specific IgG antibody	Intercept	β (Slope)	P-value	R²	Sample size
Wheat	0.3033	-0.3192	**0.0027**	0.0218	410
Soybean	0.1559	-0.2067	0.0892	0.0071	410
Codfish	0.1028	-0.2574	0.163	0.0048	410
Egg	0.3444	-0.1292	0.2017	0.004	410
Rice	0.0815	-0.1356	0.4654	0.0013	410
Shrimp	0.0402	0.1991	0.5425	0.0009	410
Corn	0.0363	0.2084	0.5695	0.0008	410
Crab	0.0255	0.1212	0.6015	0.0007	409
Beef	0.0546	-0.1612	0.7305	0.0003	410
Pork	0.0454	0.2088	0.7455	0.0003	410
Tomato	0.0556	-0.0362	0.8655	0.0001	410
Chicken	0.0525	-0.0549	0.8822	0.0001	410
Mushroom	0.0507	-0.0075	0.9727	0	410
Milk	0.0467	0.0016	0.9859	0	410

• β represents the regression coefficient (slope) from the linear regression model.

• Significant P-value is in bold and marked with an asterisk. We can add a note: * P<0.05.

### 3.6 Adult cohort characteristics and hepatic dysregulation

The underweight group skewed younger (63.3% aged 18–30 vs. 31.6% controls, *p* < 0.001) with lower mean BMI (16.53 ± 1.15 vs. 24.12 ± 4.46, *p* < 0.001). Hepatic profiling revealed elevated bilirubin abnormalities in the underweight group (total bilirubin: 10% vs. 0%, *p* = 0.007; direct bilirubin: 40% vs. 6.2%, *p* = 0.003) alongside globulin dysregulation (10% vs. 0%, *p* = 0.018) (**Supplementary Table 2**). To identify systemic drivers of these clinical patterns, we performed factor analysis on adult metabolic profiles.

### 3.7 Multifactorial drivers of adult nutritional status

**Figure 2:**
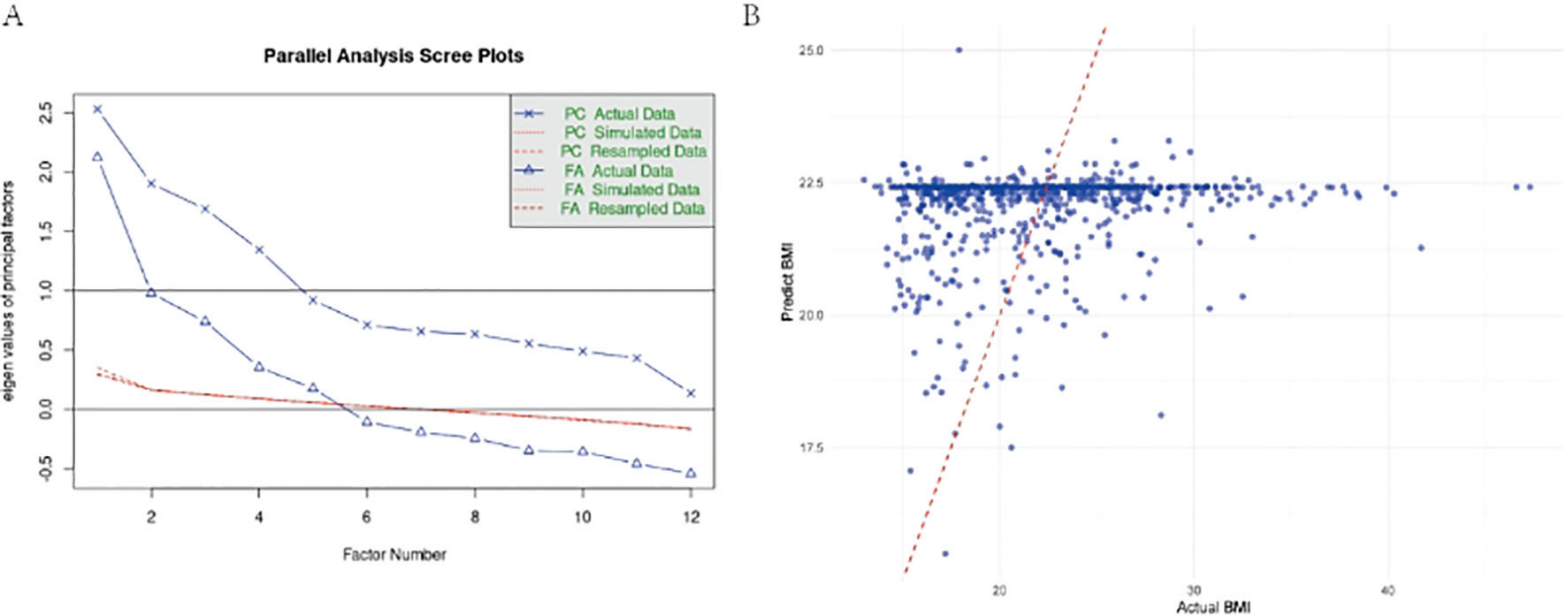
**(A)** Parallel Analysis Scree Plot for All Valid Indicators. This plot compares eigenvalues from actual data (solid line, dots) with mean eigenvalues generated from random data (dashed line, crosses) to determine the number of factors. The red line represents the eigenvalue threshold of 1 (Kaiser criterion). **(B)** Comparison of predicted vs. actual BAZ values using multivariate GLM model in adult group. Scatter plot showing the relationship between model-predicted BAZ values and actual BAZ values, with a red dashed line indicating perfect prediction.

Nine factors explained 44.3% of variance (**Figure 2A**). Hepatic function (Factor 3: *r* = 0.159, *p* < 0.001) and vitamin K status (Factor 9: *r* = 0.144, *p* < 0.001) independently predicted BMI (**Table 6**). The staple food IgG factor showed trending negative association (*r* = −0.117, *p* = 0.073), while globulin balance correlated positively (*r* = 0.125, *p* = 0.003). Prompted by the marginal association with food IgG, we specifically examined antigen-specific sensitization patterns.

**Table 6:** Factor analysis results and their correlations with BMI in adult.

Factor	Name & interpretation	Major loading indicators (Loading Value ≥ |0.4|)	Correlation with BMI
PA1	Liver Enzyme Activity	ALT (1.040), AST (0.788), γ-GGT (0.531)	0.156*
PA2	Renal Function Markers	Creatinine (0.858), Cystatin C (0.621), Uric acid (0.493)	0.114*
PA3	Grain Food Sensitivities	Soybean IgG (0.708), Rice IgG (0.624), Wheat IgG (0.546)	-0.130*
PA4	Vitamin K Metabolism	Vitamin K2 (0.628), Vitamin K1 (0.544)	0.125*
PA5	Nutritional Metabolism	Prealbumin (0.610), Uric acid (0.493)	0.126*

### 3.8 Food-specific IgG associations in adult underweight population

Soybean IgG demonstrated the strongest inverse BMI relationship (univariate β = −1.1085, *p* = 0.0003; *R*² = 1.67%), remaining significant in multivariate analysis (β = −0.9237, *p* = 0.0180) (**Figure 2B**). Nutrition problem cases showed doubled soybean sensitization rates (22.8% vs. 11.0%, OR = 2.4, *p* < 0.001). The multivariate model explained minimal BMI variance (adjusted *R*² = 0.00856, *p* = 0.114), indicating limited clinical predictive utility (**Table 7**).

**Table 7:** Multivariate linear regression analysis of food-specific IgG antibodies and their association with BMI in adult population.

Variable	Coefficient	Standard error	t-value	P-value
Soybean-specific IgG antibody	-0.9237	0.3898	-2.3698	0.018
Pork-specific IgG antibody	-0.6911	0.4632	-1.4921	0.1361
Chicken-specific IgG antibody	-1.7748	1.3596	-1.3054	0.1922
Beef-specific IgG antibody	0.8606	0.8934	0.9633	0.3357
Corn-specific IgG antibody	-0.6089	0.7144	-0.8523	0.3943
Tomato-specific IgG antibody	-0.6277	0.7898	-0.7947	0.427
Codfish-specific IgG antibody	0.4362	0.7594	0.5744	0.5659
Milk-specific IgG antibody	-0.11	0.2227	-0.494	0.6214
Wheat-specific IgG antibody	-0.1067	0.3217	-0.3317	0.7402
Mushroom-specific IgG antibody	-0.1376	0.4836	-0.2846	0.776
Shrimp-specific IgG antibody	0.154	0.7166	0.2149	0.8299
Crab-specific IgG antibody	0.1215	0.5752	0.2113	0.8327
Rice-specific IgG antibody	-0.0886	0.5845	-0.1516	0.8796
Egg-specific IgG antibody	-0.0074	0.1704	-0.0431	0.9656

But note: the original data has the variable name repeated twice in each row. We are ignoring the second one (which is the same string) and taking the first number as the coefficient.

## 4 Discussion

The observed age-dependent divergence in immune-nutrition interactions reveals distinct immunological prioritization across developmental stages. The stronger inverse association between wheat-specific IgG and body mass index z-scores in children (β = −0.357, R² = 2.18%) contrasts with the prominent soybean IgG-BMI relationship in adults (β = −0.9237, R² = 1.67%), suggesting a fundamental reorganization of dietary antigen responses with age. This shift likely reflects developmental changes in gut barrier integrity and oral tolerance mechanisms, as gut barrier maturation during childhood critically shapes immune-diet interactions (19, 20). The pediatric immune system, still refining its tolerance to dietary antigens, may exhibit heightened sensitivity to ubiquitous staples like wheat, where transient barrier immaturity amplifies IgG-mediated nutrient absorption disruptions (21). Conversely, adults’ cumulative exposure to soy—a common component of urbanized diets (Shengzheng, China)—may drive chronic low-grade inflammation through persistent antigenic stimulation, aligning with evidence linking long-term dietary patterns to immune-metabolic dysregulation (22).

These age-related patterns are further contextualized by the gut-liver axis’s evolving role in metabolic homeostasis. Pediatric ALT/AST elevations, indicative of subclinical hepatic stress, likely reflect early gut-derived inflammatory signals interfering with growth-related anabolism, a phenomenon corroborated by proteomic studies of hepatic metabolic regulation (23). In adults, bilirubin abnormalities signal advanced gut-liver axis dysfunction, potentially arising from decades of antigenic translocation and hepatic detoxification overload (24, 25). The disparity in vitamin K2 sufficiency (94.6% vs. 89.5%) underscores its dual role as a biomarker of gut absorption efficiency and hepatic activation capacity, bridging intestinal health with systemic metabolic outcomes (26, 27). This integrative role positions vitamin K2 as a critical node in the IgG-nutrition interplay, particularly in undernutrition contexts where hepatic and intestinal functions are interdependent (28).

The bidirectional IgG-nutrition relationship challenges conventional causal paradigms, particularly in underweight populations. Elevated IgG levels may emerge as both drivers and consequences of malnutrition: nutrient deficiencies impair gut barrier function, facilitating antigenic translocation and subsequent IgG elevation—a mechanism mirroring kwashiorkor-associated enteropathy (29). This reciprocity is compounded by sex-specific disparities, with male-predominant pediatric cohorts (54%) and female-dominated adult groups (74.6%) suggesting hormonal modulation of mucosal immunity. Estrogen’s known influence on gut barrier integrity and immune cell trafficking may partially explain these demographic shifts, though further investigation is needed (30, 31).

Despite these insights, the modest predictive power of individual IgG biomarkers (R² <3%) limits their standalone clinical utility, echoing challenges in dietary intervention trials for immune-mediated gastrointestinal disorders (32). Our multifactorial model, integrating hepatic and nutritional markers, substantially improved variance explanation (42.4–44.3%), emphasizing the necessity of systems-level approaches. However, the retrospective design precludes causal inference, a limitation shared by prior IgG-diet studies (33). Longitudinal studies tracking IgG dynamics during nutritional rehabilitation are imperative to disentangle causation from correlation, particularly given the potential for nutritional recovery to reverse barrier dysfunction and attenuate IgG reactivity (34).

Future research must prioritize intervention trials evaluating IgG-guided dietary modifications in undernutrition, ensuring ethical prioritization of nutritional adequacy. Combining IgG profiling with multi-omics analyses—including microbiome sequencing and gut barrier integrity markers—will clarify mechanistic pathways and identify personalized therapeutic targets (22, 25). Standardizing underweight-specific IgG reference ranges remains critical, as population-specific thresholds may explain inconsistent outcomes of elimination diets across studies (35). By elucidating IgG’s role as both a biomarker and modifiable factor in undernutrition, this work advances a precision nutrition framework that harmonizes immune and metabolic health across the lifespan.

## 5 Conclusion

This comprehensive analysis establishes food-specific IgG evaluation as a valuable adjunct tool for personalizing dietary management in underweight populations, while highlighting critical knowledge gaps requiring interdisciplinary research approaches.

## Data Availability

The raw data supporting the conclusions of this article will be made available by the authors, without undue reservation.
