# Supplementary material for: Food-specific IgG antibodies and body mass index: multivariate analysis of clinical correlations in underweight populations
Source: Front Immunol. 2025 Oct 21;16:1650705. doi: 10.3389/fimmu.2025.1650705 (PMC12583155; doi:10.3389/fimmu.2025.1650705)
Supplement: Supplementary file 1 [file SupplementaryFile1.docx]

****Supplement Table 1 . Comparison of Baseline Characteristics in Child Data by Underweight Status****

| **Indicator** | **non-underweight** | **underweight** | **p-value** |
| --- | --- | --- | --- |
| **n** | 277 | 144 |  |
| **Gender = Female (%%)** | 118 (42.6) | 71 (49.3) | 0.227 |
| **Age_Group (%%)** |  |  | 0.081 |
| 5-10 years | 88 (31.8) | 60 (41.7) |  |
| 10-15 years | 155 (56.0) | 73 (50.7) |  |
| 15-18 years | 34 (12.3) | 11 (7.6) |  |
| **BAZ (mean (SD))** | 0.92 (1.99) | -1.57 (0.39) | <0.001 |
| **ALT (%%)** |  |  | 0.007 |
| Low | 4 (8.3) | 4 (50.0) |  |
| High | 4 (8.3) | 0 (0.0) |  |
| Normal | 40 (83.3) | 4 (50.0) |  |
| **AST (%%)** |  |  | 0.777 |
| Low | 10 (20.8) | 1 (12.5) |  |
| High | 1 (2.1) | 0 (0.0) |  |
| Normal | 37 (77.1) | 7 (87.5) |  |
| **GGT (%%)** |  |  | 0.018 |
| Low | 5 (10.4) | 4 (50.0) |  |
| High | 1 (2.1) | 0 (0.0) |  |
| Normal | 42 (87.5) | 4 (50.0) |  |
| **TB (%%)** |  |  | 0.841 |
| Low | 1 (2.1) | 0 (0.0) |  |
| High | 1 (2.1) | 0 (0.0) |  |
| Normal | 46 (95.8) | 8 (100.0) |  |
| **DB = Normal (%%)** | 43 (89.6) | 8 (100.0) | 0.774 |
| **IB = Normal (%%)** | 43 (89.6) | 8 (100.0) | 0.774 |
| **UREA = Normal (%%)** | 80 (84.2) | 18 (100.0) | 0.152 |
| **CREA = Normal (%%)** | 46 (48.4) | 8 (44.4) | 0.958 |
| **UA = Normal (%%)** | 66 (69.5) | 16 (88.9) | 0.16 |
| **CysC (%%)** |  |  | 0.853 |
| Low | 1 (1.8) | 0 (0.0) |  |
| High | 6 (10.9) | 2 (12.5) |  |
| Normal | 48 (87.3) | 14 (87.5) |  |
| **TP_Status = Normal (%%)** | 36 (97.3) | 5 (83.3) | 0.644 |
| **ALB_Status = Normal (%%)** | 37 (100.0) | 6 (100.0) | - |
| **GLB_Status = Normal (%%)** | 35 (94.6) | 6 (100.0) | 1 |
| **PA_Status = Normal (%%)** | 33 (89.2) | 3 (50.0) | 0.069 |
| **VitD_Status = Normal (%%)** | 20 (7.2) | 14 (9.7) | 0.481 |
| **K2_Status (%%)** |  |  | 0.013 |
| Low | 2 (1.0) | 4 (3.6) |  |
| High | 18 (9.4) | 2 (1.8) |  |
| Normal | 171 (89.5) | 105 (94.6) |  |
| **VitD25OH_Status (%%)** |  |  | 0.017 |
| Low | 45 (23.6) | 12 (10.8) |  |
| High | 1 (0.5) | 0 (0.0) |  |
| Normal | 145 (75.9) | 99 (89.2) |  |
| **K1_Status (%%)** |  |  | 0.27 |
| Low | 4 (2.1) | 5 (4.5) |  |
| High | 7 (3.7) | 7 (6.3) |  |
| Normal | 180 (94.2) | 99 (89.2) |  |
| **Milk-specific IgG antibody (%%)** |  |  | 0.381 |
| Level 0 | 51 (18.5) | 30 (20.8) |  |
| Level 1 | 59 (21.5) | 28 (19.4) |  |
| Level 2 | 83 (30.2) | 34 (23.6) |  |
| Level 3 | 82 (29.8) | 52 (36.1) |  |
| **Egg-specific IgG antibody (%%)** |  |  | 0.266 |
| Level 0 | 29 (10.5) | 11 (7.6) |  |
| Level 1 | 23 (8.4) | 14 (9.7) |  |
| Level 2 | 72 (26.2) | 28 (19.4) |  |
| Level 3 | 151 (54.9) | 91 (63.2) |  |
| **Wheat-specific IgG antibody (%%)** |  |  | 0.001 |
| Level 0 | 149 (54.2) | 60 (41.7) |  |
| Level 1 | 76 (27.6) | 36 (25.0) |  |
| Level 2 | 42 (15.3) | 33 (22.9) |  |
| Level 3 | 8 (2.9) | 15 (10.4) |  |
| **Rice-specific IgG antibody (%%)** |  |  | 0.324 |
| Level 0 | 224 (81.5) | 114 (79.2) |  |
| Level 1 | 45 (16.4) | 22 (15.3) |  |
| Level 2 | 4 (1.5) | 6 (4.2) |  |
| Level 3 | 2 (0.7) | 2 (1.4) |  |
| **Corn-specific IgG antibody (%%)** |  |  | 0.325 |
| Level 0 | 260 (94.5) | 135 (93.8) |  |
| Level 1 | 12 (4.4) | 9 (6.2) |  |
| Level 2 | 3 (1.1) | 0 (0.0) |  |
| **Soy-specific IgG antibody (%%)** |  |  | 0.102 |
| Level 0 | 190 (69.1) | 82 (56.9) |  |
| Level 1 | 57 (20.7) | 41 (28.5) |  |
| Level 2 | 17 (6.2) | 12 (8.3) |  |
| Level 3 | 11 (4.0) | 9 (6.2) |  |
| **Cod-specific IgG antibody (%%)** |  |  | 0.235 |
| Level 0 | 240 (87.3) | 115 (79.9) |  |
| Level 1 | 26 (9.5) | 20 (13.9) |  |
| Level 2 | 7 (2.5) | 7 (4.9) |  |
| Level 3 | 2 (0.7) | 2 (1.4) |  |
| **Crab-specific IgG antibody (%%)** |  |  | 0.266 |
| Level 0 | 253 (92.3) | 129 (89.6) |  |
| Level 1 | 15 (5.5) | 11 (7.6) |  |
| Level 2 | 3 (1.1) | 4 (2.8) |  |
| Level 3 | 3 (1.1) | 0 (0.0) |  |
| **Tomato-specific IgG antibody (%%)** |  |  | 0.578 |
| Level 0 | 240 (87.3) | 122 (84.7) |  |
| Level 1 | 26 (9.5) | 18 (12.5) |  |
| Level 2 | 7 (2.5) | 4 (2.8) |  |
| Level 3 | 2 (0.7) | 0 (0.0) |  |
| **Mushroom-specific IgG antibody (%%)** |  |  | 0.049 |
| Level 0 | 234 (85.1) | 128 (88.9) |  |
| Level 1 | 30 (10.9) | 15 (10.4) |  |
| Level 2 | 11 (4.0) | 0 (0.0) |  |
| Level 3 | 0 (0.0) | 1 (0.7) |  |
| **Shrimp-specific IgG antibody (%%)** |  |  | 0.734 |
| Level 0 | 267 (97.1) | 141 (97.9) |  |
| Level 1 | 3 (1.1) | 2 (1.4) |  |
| Level 2 | 3 (1.1) | 1 (0.7) |  |
| Level 3 | 2 (0.7) | 0 (0.0) |  |
| **Beef-specific IgG antibody (%%)** |  |  | 0.441 |
| Level 0 | 270 (98.2) | 139 (96.5) |  |
| Level 1 | 4 (1.5) | 3 (2.1) |  |
| Level 2 | 1 (0.4) | 2 (1.4) |  |
| **Pork-specific IgG antibody (%%)** |  |  | 0.555 |
| Level 0 | 271 (98.5) | 141 (97.9) |  |
| Level 1 | 3 (1.1) | 3 (2.1) |  |
| Level 2 | 1 (0.4) | 0 (0.0) |  |
| **Chicken-specific IgG antibody (%%)** |  |  | 0.471 |
| Level 0 | 265 (96.4) | 135 (93.8) |  |
| Level 1 | 8 (2.9) | 7 (4.9) |  |
| Level 2 | 2 (0.7) | 2 (1.4) |  |

### Note: Abbreviations: ALT, alanine aminotransferase; AST, aspartate aminotransferase; BAZ, BMI-for-age z-score; CREA, creatinine; CysC, cystatin C; DB, direct bilirubin; GGT, gamma-glutamyl transferase; GLB, globulin; IB, indirect bilirubin; IgG, immunoglobulin G; K1, vitamin K1; K2, vitamin K2; PA, prealbumin; TB, total bilirubin; TP, total protein; UA, uric acid; VitD, total vitamin D; VitD25OH, 25-hydroxy vitamin D.

****Supplementary Table 2. Comparison of Baseline Characteristics in Adult Data by Underweight Status****

| **Indicator** | **Non-Nutrition Issues Group** | **Nutrition Issues Group** | **p-value** |
| --- | --- | --- | --- |
| **n** | 598 | 218 |  |
| **Age_Group (%%)** |  |  | <0.001 |
| ‣ 18-30 years | 189 (31.6) | 138 (63.3) |  |
| ‣ 31-40 years | 232 (38.8) | 46 (21.1) |  |
| ‣ 41-50 years | 100 (16.7) | 14 (6.4) |  |
| ‣ 51-60 years | 62 (10.4) | 11 (5.0) |  |
| ‣ >60 years | 15 (2.5) | 9 (4.1) |  |
| **Gender = Female (%%)** | 446 (74.6) | 145 (66.5) | 0.028 |
| **BMI (mean (SD))** | 24.12 (4.46) | 16.53 (1.15) | <0.001 |
| **ALT (%%)** |  |  | 0.192 |
| ‣ Low | 8 (8.2) | 2 (20.0) |  |
| ‣ High | 8 (8.2) | 2 (20.0) |  |
| ‣ Normal | 81 (83.5) | 6 (60.0) |  |
| **AST (%%)** |  |  | 0.215 |
| ‣ Low | 23 (23.7) | 1 (10.0) |  |
| ‣ High | 6 (6.2) | 2 (20.0) |  |
| ‣ Normal | 68 (70.1) | 7 (70.0) |  |
| **GGT (%%)** |  |  | 0.636 |
| ‣ Low | 10 (10.3) | 1 (10.0) |  |
| ‣ High | 8 (8.2) | 0 (0.0) |  |
| ‣ Normal | 79 (81.4) | 9 (90.0) |  |
| **TB (%%)** |  |  | 0.007 |
| ‣ Low | 1 (1.0) | 0 (0.0) |  |
| ‣ High | 0 (0.0) | 1 (10.0) |  |
| ‣ Normal | 96 (99.0) | 9 (90.0) |  |
| **DB = Normal (%%)** | 91 (93.8) | 6 (60.0) | 0.003 |
| **IB (%%)** |  |  | 0.005 |
| ‣ Low | 8 (8.3) | 0 (0.0) |  |
| ‣ High | 0 (0.0) | 1 (10.0) |  |
| ‣ Normal | 88 (91.7) | 9 (90.0) |  |
| **UREA = Normal (%%)** | 184 (91.1) | 20 (90.9) | 1 |
| **CREA (%%)** |  |  | 0.404 |
| ‣ Low | 43 (21.2) | 2 (9.1) |  |
| ‣ High | 16 (7.9) | 2 (9.1) |  |
| ‣ Normal | 144 (70.9) | 18 (81.8) |  |
| **UA (%%)** |  |  | 0.038 |
| ‣ Low | 2 (1.0) | 0 (0.0) |  |
| ‣ High | 59 (29.2) | 1 (4.5) |  |
| ‣ Normal | 141 (69.8) | 21 (95.5) |  |
| **CysC (%%)** |  |  | 0.879 |
| ‣ Low | 2 (1.5) | 0 (0.0) |  |
| ‣ High | 8 (6.0) | 1 (5.9) |  |
| ‣ Normal | 124 (92.5) | 16 (94.1) |  |
| **GFR = Normal (%%)** | 12 (44.4) | 0 (0.0) | 1 |
| **TP_Status = Normal (%%)** | 76 (97.4) | 10 (100.0) | 1 |
| **ALB_Status = Normal (%%)** | 77 (98.7) | 10 (100.0) | 1 |
| **GLB_Status (%%)** |  |  | 0.018 |
| ‣ Low | 1 (1.3) | 0 (0.0) |  |
| ‣ High | 0 (0.0) | 1 (10.0) |  |
| ‣ Normal | 77 (98.7) | 9 (90.0) |  |
| **PA_Status = Normal (%%)** | 73 (93.6) | 8 (80.0) | 0.382 |
| **VitD_Status = Normal (%%)** | 28 (4.7) | 11 (5.0) | 0.976 |
| **K2_Status (%%)** |  |  | 0.336 |
| ‣ Low | 3 (0.8) | 3 (2.3) |  |
| ‣ High | 41 (10.3) | 12 (9.2) |  |
| ‣ Normal | 354 (88.9) | 116 (88.5) |  |
| **VitD25OH_Status (%%)** |  |  | 0.212 |
| ‣ Low | 176 (44.1) | 56 (42.4) |  |
| ‣ High | 0 (0.0) | 1 (0.8) |  |
| ‣ Normal | 223 (55.9) | 75 (56.8) |  |
| **K1_Status (%%)** |  |  | 0.048 |
| ‣ Low | 201 (33.6) | 88 (40.4) |  |
| ‣ High | 57 (9.5) | 11 (5.0) |  |
| ‣ Normal | 340 (56.9) | 119 (54.6) |  |
| **Milk-specific IgG antibody (%%)** |  |  | 0.031 |
| ‣ Level 0 | 406 (68.7) | 130 (60.5) |  |
| ‣ Level 1 | 89 (15.1) | 32 (14.9) |  |
| ‣ Level 2 | 59 (10.0) | 37 (17.2) |  |
| ‣ Level 3 | 37 (6.3) | 16 (7.4) |  |
| **Egg-specific IgG antibody (%%)** |  |  | 0.193 |
| ‣ Level 0 | 208 (35.2) | 65 (30.2) |  |
| ‣ Level 1 | 129 (21.8) | 41 (19.1) |  |
| ‣ Level 2 | 130 (22.0) | 50 (23.3) |  |
| ‣ Level 3 | 124 (21.0) | 59 (27.4) |  |
| **Wheat-specific IgG antibody (%%)** |  |  | 0.228 |
| ‣ Level 0 | 497 (84.1) | 169 (78.6) |  |
| ‣ Level 1 | 55 (9.3) | 26 (12.1) |  |
| ‣ Level 2 | 28 (4.7) | 12 (5.6) |  |
| ‣ Level 3 | 11 (1.9) | 8 (3.7) |  |
| **Rice-specific IgG antibody (%%)** |  |  | 0.01 |
| ‣ Level 0 | 555 (93.9) | 190 (88.4) |  |
| ‣ Level 1 | 27 (4.6) | 15 (7.0) |  |
| ‣ Level 2 | 9 (1.5) | 8 (3.7) |  |
| ‣ Level 3 | 0 (0.0) | 2 (0.9) |  |
| **Corn-specific IgG antibody (%%)** |  |  | 0.102 |
| ‣ Level 0 | 566 (95.8) | 201 (93.5) |  |
| ‣ Level 1 | 21 (3.6) | 10 (4.7) |  |
| ‣ Level 2 | 4 (0.7) | 2 (0.9) |  |
| ‣ Level 3 | 0 (0.0) | 2 (0.9) |  |
| **Soy-specific IgG antibody (%%)** |  |  | <0.001 |
| ‣ Level 0 | 526 (89.0) | 166 (77.2) |  |
| ‣ Level 1 | 38 (6.4) | 29 (13.5) |  |
| ‣ Level 2 | 20 (3.4) | 14 (6.5) |  |
| ‣ Level 3 | 7 (1.2) | 6 (2.8) |  |
| **Cod-specific IgG antibody (%%)** |  |  | 0.369 |
| ‣ Level 0 | 567 (95.9) | 207 (96.3) |  |
| ‣ Level 1 | 19 (3.2) | 6 (2.8) |  |
| ‣ Level 2 | 5 (0.8) | 1 (0.5) |  |
| ‣ Level 3 | 0 (0.0) | 1 (0.5) |  |
| **Crab-specific IgG antibody (%%)** |  |  | 0.622 |
| ‣ Level 0 | 558 (94.7) | 203 (94.4) |  |
| ‣ Level 1 | 25 (4.2) | 10 (4.7) |  |
| ‣ Level 2 | 3 (0.5) | 0 (0.0) |  |
| ‣ Level 3 | 2 (0.3) | 2 (0.9) |  |
| **Tomato-specific IgG antibody (%%)** |  |  | 0.784 |
| ‣ Level 0 | 565 (95.8) | 205 (95.3) |  |
| ‣ Level 1 | 20 (3.4) | 7 (3.3) |  |
| ‣ Level 2 | 5 (0.8) | 3 (1.4) |  |
| **Mushroom-specific IgG antibody (%%)** |  |  | 0.4 |
| ‣ Level 0 | 549 (92.9) | 192 (89.7) |  |
| ‣ Level 1 | 30 (5.1) | 17 (7.9) |  |
| ‣ Level 2 | 11 (1.9) | 4 (1.9) |  |
| ‣ Level 3 | 1 (0.2) | 1 (0.5) |  |
| **Shrimp-specific IgG antibody (%%)** |  |  | 0.334 |
| ‣ Level 0 | 553 (93.6) | 203 (94.4) |  |
| ‣ Level 1 | 36 (6.1) | 10 (4.7) |  |
| ‣ Level 2 | 2 (0.3) | 1 (0.5) |  |
| ‣ Level 3 | 0 (0.0) | 1 (0.5) |  |
| **Beef-specific IgG antibody (%%)** |  |  | 0.594 |
| ‣ Level 0 | 583 (98.8) | 214 (99.5) |  |
| ‣ Level 1 | 3 (0.5) | 0 (0.0) |  |
| ‣ Level 2 | 2 (0.3) | 0 (0.0) |  |
| ‣ Level 3 | 2 (0.3) | 1 (0.5) |  |
| **Pork-specific IgG antibody (%%)** |  |  | 0.135 |
| ‣ Level 0 | 585 (99.0) | 212 (98.6) |  |
| ‣ Level 1 | 3 (0.5) | 0 (0.0) |  |
| ‣ Level 2 | 1 (0.2) | 2 (0.9) |  |
| ‣ Level 3 | 2 (0.3) | 0 (0.0) |  |
| ‣ Level 10 | 0 (0.0) | 1 (0.5) |  |
| **Chicken-specific IgG antibody (%%)** |  |  | 0.142 |
| ‣ Level 0 | 586 (99.2) | 213 (99.1) |  |
| ‣ Level 1 | 4 (0.7) | 0 (0.0) |  |
| ‣ Level 2 | 1 (0.2) | 2 (0.9) |  |
